# Ryanodine receptors, a family of intracellular calcium ion channels, are expressed throughout early vertebrate development

**DOI:** 10.1186/1756-0500-4-541

**Published:** 2011-12-14

**Authors:** Houdini HT Wu, Caroline Brennan, Rachel Ashworth

**Affiliations:** 1 School of Biological and Chemical Sciences, Queen Mary University of London, Mile End Road, London E1 4NS, UK

## Abstract

**Background:**

Calcium signals ([Ca^2+^]_i_) direct many aspects of embryo development but their regulation is not well characterised. Ryanodine receptors (RyRs) are a family of intracellular Ca^2+ ^release channels that control the flux of Ca^2+ ^from internal stores into the cytosol. RyRs are primarily known for their role in excitation-contraction coupling in adult striated muscle and *ryr *gene mutations are implicated in several human diseases. Current evidence suggests that RyRs do not have a major role to play prior to organogenesis but regulate tissue differentiation.

**Findings:**

The sequences of the five zebrafish *ryr *genes were confirmed, their evolutionary relationship established and the primary sequences compared to other vertebrates, including humans. RyRs are differentially expressed in slow (*ryr1a*), fast (*ryr3*) and both types (*ryr1b*) of developing skeletal muscle. There are two *ryr2 *genes (*ryr2a *and *ryr2b*) which are expressed exclusively in developing CNS and cardiac tissue, respectively. In addition, *ryr3 *and *ryr2a *mRNA is detectable in the initial stages of development, prior to embryonic axis formation.

**Conclusions:**

Our work reveals that zebrafish *ryr *genes are differentially expressed throughout the developing embryo from cleavage onwards. The data suggests that RyR-regulated Ca^2+ ^signals are associated with several aspects of embryonic development, from organogenesis through to the differentiation of the musculoskeletal, cardiovascular and nervous system. These studies will facilitate further work to explore the developmental function of RyRs in each of these tissue types.

## Background

Transient changes in the concentration of intracellular calcium ions ([Ca^2+^]_i_) act as a powerful signal that is crucial for the establishment of form and function in the embryo. Detailed imaging studies have revealed that the spatial and temporal organisation of Ca^2+ ^signals during embryogenesis are associated with many of the major phases of development, from early cell division to the differentiation of tissues. Despite their importance little is known about the generation and regulation of embryonic Ca^2+ ^signals. A comprehensive understanding of the pathways that regulate [Ca^2+^]_i _during development is essential to understand the functional relevance of these signals in the embryo.

Ryanodine receptors (RyR) are a family of intracellular Ca^2+ ^release channels that regulate the entry of Ca^2+ ^into the cytosol from the intracellular organelles (the endoplasmic and sarcoplasmic reticulum). The RyR is a large homotetrameric protein (approximately 2,200 kDa), each subunit is comprised of a large N-terminal cytoplasmic domain which modulates the gating of the channel, as well as luminal and transmembrane spanning (TM) domains. In mammals, there are three distinct *ryr *genes (*ryr1*, *ryr2 *and *ryr3*) that encode three differentially expressed RyR proteins. RyR1 and RyR2 are expressed predominantly in skeletal and cardiac muscle respectively, whilst RyR3 is found in many tissues at relatively low levels [1]. The primary role of the RyR is to increase [Ca^2+^]_i _during excitation-contraction coupling (E-C coupling) in both skeletal and cardiac muscle. In humans, mutations in the *ryr1 *and *ryr2 *genes cause skeletal myopathies and cardiac disease respectively [2]. The generation of mouse knockout lines has provided some insight into the role of the receptors in the developing tissues of intact animals. Homozygous mice from *ryr1*^(−/−) ^(*skrrm1*) and *ryr2^(−/−) ^*knockout lines display gross morphological defects in either the skeletal muscle (*ryr1*) or heart tube (*ryr2*) and die at the perinatal or embryonic day 10 (E10) stages respectively [3,4]. In contrast, *ryr3^(−/−) ^*knockout mice appear to have no gross developmental defects and evidence suggests that RyR3 act to augment the [Ca^2+^]_i _response of the other RyR isoforms in striated muscle [5,6]. The observation that RyR expression does not occur until relatively late in mammalian development [7], coupled with the fact that knock out lines are not lethal at very early stages has been interpreted as indicating that RyRs do not function during initial development.

The zebrafish has been used extensively as a model for vertebrate development. The rapid development *ex utero *and embryonic transparency has proved advantageous for imaging the spatial and temporal organisation of Ca^2+ ^signals. These signals are involved in many of the early embryonic events; the initiation of fertilisation (0 hours post fertilisation, hpf), the early cell divisions associated with the cleavage period (up to 2 hpf) and the more extensive cellular rearrangements that occur in the blastula period (up to 5 hpf) (as reviewed in [8]). Evidence suggests that the release of Ca^2+ ^from intracellular stores via the phosphatidylinositol (PI) signalling pathway is largely responsible for these early transient changes in [Ca^2+^]_i _[9]. RyRs have not been implicated in very early developmental events and their expression during these stages has not been documented. Fluxes in the levels of embryonic [Ca^2+^]_i _continue to occur throughout gastrulation (up to 10 hpf) (as reviewed in [8]). Initially changes in [Ca^2+^]_i _occur as localised events but, as gastrulation progresses, co-ordinated waves of Ca^2+ ^signalling appear across the embryo. These later signals are proposed to coordinate a wide range of cellular movements (epiboly, involution, convergence and extension) that give rise to the embryonic body plan. Release of Ca^2+ ^from intracellular stores via the PI signalling pathway is again implicated at this stage; however, the contribution to gastrulation from other Ca^2+ ^signalling pathways remains undefined. Finally, the segmentation period (from 10 up to 24 hpf) is characterised by organogenesis and the emergence of the body systems. The Ca^2+ ^signals that occur during segmentation are again more localised and typically associated with developing tissues. Transient changes in [Ca^2+^]_i _have been recorded within the nervous system, somites and cardiac tissue [10-12]. Several studies in zebrafish have shown that inhibition of ryanodine receptor function, using both pharmacological and genetic inhibitors, leads to impaired excitation-contraction coupling and gross morphological defects in the skeletal muscle, suggestive of a role in the development of this tissue [11,13,14].

This study set out to acquire a more comprehensive understanding of ryanodine receptor expression in early vertebrate development, using the zebrafish as an *in vivo *model. Our initial work confirmed the sequence of the five zebrafish *ryr *genes, established their evolutionary relationship to those in other vertebrate species and provided a direct comparison between the structural features of the primary protein sequences found in the zebrafish and mammals. An overview of *ryr *gene expression during zebrafish embryogenesis will inform work aimed at establishing the developmental significance of this family of Ca^2+^-release channels. Therefore we conducted a comprehensive temporal and spatial analysis of *ryr *mRNA expression in the embryo using a combination of semi-quantitative PCR and wholemount *in situ *hybridisation. We observed strong maternal expression of *ryr *mRNA (*ryr3 and ryr2a*) during the cleavage and blastula periods suggestive of a novel role in early development. At 24 hours post fertilisation (hpf) *ryr1a*, *ryr1b *and *ryr3 *are expressed in skeletal muscle, whereas *ryr2a *is localized to the central nervous system (CNS) and *ryr2b *is found exclusively in the cardiac muscle. Our study suggests that RyR channels have a role in early development prior to organogenesis as well as in the differentiation of different cell types.

## Methods

### Animal procedures

Wildtype (WT) zebrafish strains (*Tubingen *and *Tupfel long fin*) were bred and raised in-house at the zebrafish facility of Queen Mary College, University of London, UK, as described previously [15]. *Smoothened *(*smo*) mutants were received as a gift from Prof. Simon Hughes (King's College London, UK). Embryos were collected by natural spawning and staged according to Kimmel and colleagues [16], given in the text as standard developmental time at 28.5°C (hours post fertilisation, hpf). Work on zebrafish embryos (prior to independent feeding) is exempt under the U.K. Animals (Scientific Procedures) Act 1986 and does not require ethical approval.

### Genomic analysis and gene prediction of *ryr *genes

**Figure 1:**
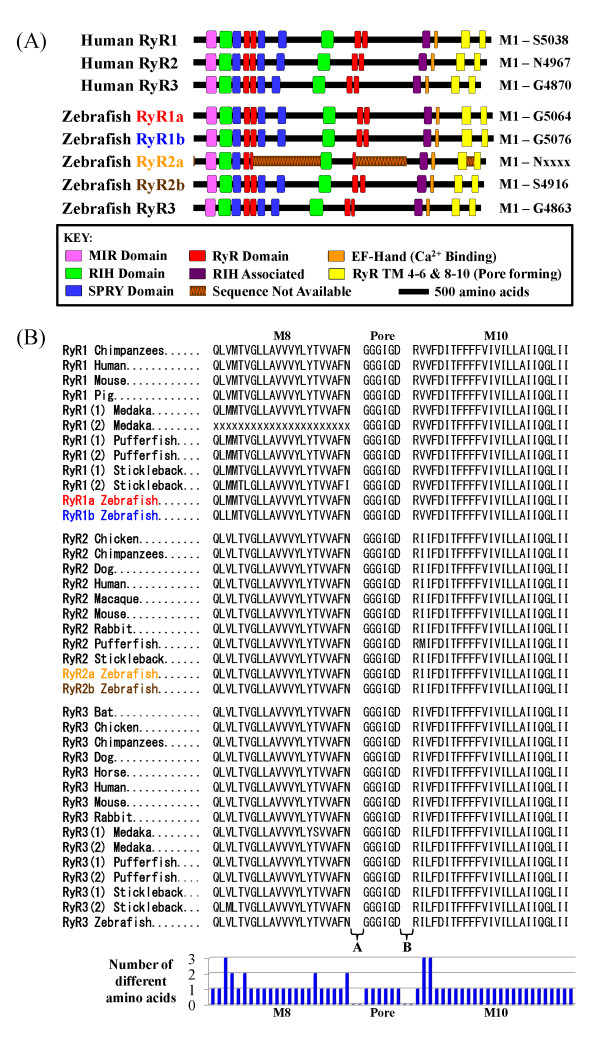
**Analysis of the primary sequence of the zebrafish RyRs**. **(a) **Schematic representation of the protein sequence of zebrafish RyRs compared to the human isoforms. The figure shows the three human RyRs at the top and the five zebrafish RyRs below. Sequence equivalent to MIR and RIH domains identified in all five zebrafish RyRs. Three SPRY domains, four copies of the RyR repeated domain, a single copy of the RIH associated domain and a putative EF hand domain were also identified within the zebrafish RyRs. Transmembrane domains (TM 4-6) and putative pore forming regions (TM 8-10) were also present. **(b) **Alignment of 36 RyR orthologues to show the sequences of the pore and transmembrane regions (M8, pore, M10). Letters A and B indicate regions not shown in alignment. Bar graph indicates the number of different amino acids among the different isoforms at the indicated residues.

To identify zebrafish *ryr *genes encoding RyRs as described in Hirata and colleagues [13], protein sequences corresponding to the human RyR family [http://www.ensembl.org/Homosapiens/familyview?family=ENSF00000000736] were used as template for searching in GenBank and the zebrafish genome resources at Ensembl http://www.ensembl.org/Danio_rerio/; Zv7 Ensembl assembly using a protein tBLASTn approach. Several hits were identified, which were then compared to the 3'-UTR sequence of the EST to identify the corresponding genomic region. The gene predictions program, Genwise (http://www.ebi.ac.uk/Wise2/) was applied to reveal genomic contigs and full sequence relating to the *ryr1a*, *ryr2a*, *ryr2b *and *ryr3 *in zebrafish (Table 1). For *ryr1a *and *ryr3*, based on the tBLASTn result, the Accession numbers (Table 2) and annotated gene sequences for these isoforms were selected. For *ryr2a*, owing to the limitations to the resources available on the database, 5 contigs (NA_1034, NA_1216, NA_3083, NA_1397 and NA_1713) were identified with nucleotide similarity to the human *ryr2*, based on the approach described above. Bioinformatics database search approaches using human *ryr2 *did not reveal any fully annotated *ryr2b *gene sequences in zebrafish. However, database blast results located a gene (EntrezGene Name LOC568506 on chromosome 17:18.7 m) encoding 805 residues. The partial *ryr2b *gene located in a 19.98 kb region in the Ensembl Zv7 assembly shared very limited conserved symmetry with the human *ryr2*. An alignment approach revealed that the zebrafish *ryr2b *gene was reverse transcribed and the region of 19.98 kb encoding 805 residues at the C-terminus end shows high peptide identity to the human RyR2 C-terminus. To help to find the missing large N-terminal cytoplasmic domain of the RyR2b peptide, Genscan features on Ensembl Zv7 assembly were employed to reveal an extra 1014 residues, giving a total of 1819 residues from the C-terminal end of the peptide. In order to search further for the missing portion of the RyR2 peptide towards the N-terminus, a genomic region of 107,783 bp spanning the C-terminal end of *ryr2b *gene to the start of neighbouring actn2 gene [Ensembl: ENSDARG00000071090], was reverse complemented using REQSEV program http://bioweb.pasteur.fr/seqanal/interfaces/revseq.html before performing a pairwise BLAST alignment with *Gasterosteus aculeatus *(stickleback) RyR2 peptide sequence on NCBI (http://www.ncbi.nlm.nih.gov/blast/bl2seq/wblast2.cgi) to confirm its sequence identity. Based on the alignment result, the full length zebrafish RyR2b peptide was first constructed with reference to *Takifugu rubripes *(pufferfish) and stickleback RyR2 peptide sequences using the Genewise program (http://www.ebi.ac.uk/Wise2/). The complete RyR2b peptide was finalised by the alignment of the translated genomic region using SHOWORF program http://bioweb.pasteur.fr/seqanal/interfaces/showorf.html. The sequence for the *ryr2b *gene was deposited in EMBL Nucleotide Sequence Database (Accession no. FR822741). For *ryr1b*, sequence was obtained via the NCBI CoreNucleotide search website [NCBI - AB247454] and isoform-specific PCR primers (set 3) were used, as described previously [13]. Isoform-specific PCR primers for *ryr1a*, *ryr2a*, *ryr2b *and *ryr3 *were designed by retrieving the nucleotide sequences based on divergent regions of the RyRs identified from peptide sequence (Figure 1). These regions were re-confirmed by both BLAST against different databases (NCBI nucleotide blast, Ensembl and ZFIN) and DNA sequencing.

**Table 1:** Summary of the *ryr *genes identified in zebrafish and their identity to the human orthologues.

Genes	cDNA length (bp)	Numbers of Exons	Genome Location	Coding Region (aa)	Identity to Human isoforms#
ryr1a	15,195	108	Chromosome 10: 29.76 m	5,064	76%

ryr1b	15,231	100*	Chromosome 18: 36.78 m	5,076	76%

ryr2a	N/A	N/A	5 contigs so far; scaffolds	2,829	86%

ryr2b	14,748	121*	Chromosome 17: 18.70 m	4,916	63%

ryr3	15,122	98	Chromosome 20: 38.11 m	4,863	77%

The zebrafish *ryr1a*, ryr1b, ryr2b and ryr3 genes are composed of 108, 100, 121 and 98 exons, and encode proteins of 5064, 5076, 4916 and 4870 amino acids (aa), respectively (Data from Zv7 Ensembl and BLAT Search). To date, five genomic contigs were identified for ryr2a, which spans 2829 amino acids, based on a protein tBLASTn searching approach using human RyR2 peptide

* = BLAT RESULTS and # = ClustalW Alignment Score

**Table 2:** Sources of ryanodine receptor sequences used for phylogenetic analysis

RyRs	Species	Transcripts	Peptides
RyR1	Chimpanzee	ENSPTRT00000020262	ENSPTRP00000018741

RyR1	Human	ENST00000359596	ENSP00000352608

RyR1	Mouse	ENSMUST00000032813	ENSMUSP00000032813

RyR1	Pig	NP_001001534	

RyR1	Rabbit	P11716	

RyR1_(1)	Medaka	ENSORLT00000008002	ENSORLP00000008001

RyR1_(2)		ENSORLT00000001305	ENSORLP00000001304

RyR1_(1)	Pufferfish	ENSTRUT00000039120	ENSTRUP00000038980

RyR1_(2)		ENSTRUT00000043571	ENSTRUP00000043426

RyR1_(1)	Stickleback	ENSGACT00000017168	ENSGACP00000017134

RyR1_(2)		ENSGACT00000027379	ENSGACP00000027327

RyR1a	Zebrafish	ENSDART00000036015	ENSDARP00000032856

RyR1b		AB247454	

RyR2	Chicken	ENSGALT00000017582	ENSGALP00000017561

RyR2	Chimpanzee	ENSPTRT00000003929	ENSPTRP00000003630

RyR2	Dog	ENSCAFT00000015962	ENSCAFP00000014764

RyR2	Human	ENST00000360064	ENSP00000353174

RyR2	Macaque	ENSMMUT00000001512	ENSMMUP00000001423

RyR2	Mouse	ENSMUST00000021750	ENSMUSP00000021750

RyR2	Rabbit	P30957	

RyR2	Pufferfish	GeneScan	

RyR2	Stickleback	ENSGACT00000020252	ENSGACP00000020213

RyR2b	Zebrafish	EMBL Nucleotide Sequence Database (Accession no. FR822741)	

RyR3	Chicken	ENSGALT00000005352	ENSGALP00000005342

RyR3	Chimpanzee	ENSPTRT00000049180	ENSPTRP00000041923

RyR3	Dog	ENSCAFT00000012974	ENSCAFP00000012007

RyR3	Horse	ENSECAT00000016127	ENSECAP00000013008

RyR3	Human	ENST00000362047	ENSP00000354852

RyR3	Mouse	ENSMUST00000091818	ENSMUSP00000089426

RyR3_(1)	Medaka	ENSORLT00000022371	ENSORLP00000022370

RyR3_(2)		ENSORLT00000021133	ENSORLP00000021132

RyR3_(1)	Pufferfish	ENSTRUT00000046340	ENSTRUP00000046185

RyR3_(2)		ENSTRUT00000036458	ENSTRUP00000036327

RyR3_(1)	Stickleback	ENSGACT00000008056	ENSGACP00000008037

RyR3_(2)		ENSGACT00000010590	ENSGACP00000010568

RyR3	Zebrafish	ENSDART00000046553	ENSDARP00000046552

### Phylogenetic Analysis

Protein sequences equivalent to the RIH_assoc (pfam08454) and RR_TM4-6 (pfam06459) domains in the five zebrafish RyR protein sequences (RyR1a, RyR1b, partial RyR2a, RyR2b and RyR3) and other well characterised vertebrates RyR homologue sequences were extracted from Ensembl and GenBank databases (Table 2). The RyR sequences were pre-aligned using the ClustalW alignment program to remove any gaps generated within the sequences, followed by a multiple RyR sequence alignment using the T-coffee program available from the European Bioinformatics Institute webpage (http://www.ebi.ac.uk/Tools/t-coffee/index.html) with the default parameters. The output from the multiple sequence alignment result obtained from T-coffee was used subsequently as the template for the generation of a Guide Tree using the equal angle method available on the SplitsTree4 program with its default parameters (http://www.splitstree.org/for free download).

### Semi-quantitative end-point PCR

Total RNA was isolated from nine stages of zebrafish development: 4- to 128-cell stage (1-2.25 hpf), 50%-epiboly stage (5.3 hpf), 100%-epiboly stage (10 hpf), 3- to 6-somite stage (11-12 hpf), 18-somite stage (18 hpf), 24 hpf, 72 hpf, 144 hpf stages and a 6-month old adult, using TRIZOL^® ^Reagent (Invitrogen). Total RNA (5 μg) was subjected to 30 minutes treatment with DNase I (Promega) at 37°C prior to cDNA synthesis performed using a Cloned AMV First-Strand cDNA Synthesis kit (Invitrogen) with random hexamers. For semi-quantitative end-point PCR, 1.5 μg of cDNA templates made from the developmental series were used alongside with the five sets of isoform-specific oligonucleotide primer pairs for amplification and normalised against the expression of *β-actin *(Table 3).

**Table 3:** Primer sequences used for PCR and WISH protocols

Genes	(A) PCR Primers (5' to 3')	(B) WISH Primers (5' to 3')
*ryr1a *F	AAAGATCATGCCCTCAGCATGC	

*ryr1a *R	TTGGAATCTCCGCCTCCGAGTA	

*ryr1b *F	CCTCCTCCTGGTTATGCACCATGTTATGAG	

*ryr1b *R	TCAATGTGGTTGTGATCAGTGGGAACGGGA	

*ryr2a *F	GAAGATGGCGAGAAGAAACCTG	ATGTCTGACGCCGGTGAACGAT

*ryr2a *R	TGCAGCATCATTGTTGTCCGTTG	CAGCACAAAGTGAACAGTGACGG

*ryr2b *F	TCTAAATTCAGCCACCATGATGTGAGC	

*ryr2b *R	TGTTGCTCTCATGTACTCGCCC	

*ryr3 *F	AAGGAAAAGAATGAGTCGGGC	

*ryr3 *R	CACGCATCTTTTCTTTGGGA	

### Whole mount in situ hybridisation

*In situ *hybridisation of whole-mounted zebrafish embryos was performed as described previously [17]. Briefly, *ryr1a*, *ryr1b*, *ryr2a*, *ryr2b *and *ryr3 *sense and anti-sense digoxygenin (DIG) labelled RNA probes covering 1,405 bp, 1,361 bp, 1,313 bp, 1,266 bp and 1,549 bp, were generated (Table 3). Briefly recombinant vector templates (pGEM-T Easy-*ryrs*) were linearised with an appropriate restriction enzyme and then subjected to phenol/chloroform purification and alcohol precipitation. Purified, linear DNA (0.5 μg) was used for the *in vitro *transcription reaction (a 20 μl reaction contained 1X Transcription Buffer (Invitrogen), 2 μl DTT (0.1 M), 2 μl nucleotide mix (1 mM GTP, 1 mM ATP, 1 mM CTP, 0.65 mM UTP and 0.35 mM DIG-11-UTP), 50U placental ribonuclease inhibitor and 10U of RNA polymerase). The mixture was incubated at 37°C for 4 hours before the removal of the original DNA template by incubating with 2U of DNase at 37°C for 1 hour. DIG-labelled cardiac myosin light chain 2 (*cmlc2*;[18]), myogenic differentiation (*myoD*;[19]), *nkx2.5 *([20]) and fluorescein-11-UTP labelled myosin heavy chain 1 (*myhz1*;[21]) anti-sense probes were also prepared under the same approach and used as positive markers.

Zebrafish embryos were fixed in 4% paraformaldehyde (PFA)/phosphate buffered Triton X100 (PBT) at 4°C overnight or at room temperature (RT) for 2 hours, then washed in 100% methanol and incubated at −20°C for at least 30 minutes. Embryos were rehydrated in a methanol gradient and treated with 10 μg/ml of Proteinase K/PBT for 1 to 20 minutes at RT depending on their stages prior to fixation in 4% PFA/PBT for 20 minutes at RT (embryos before or at 50% epiboly stage were not treated with Protease K or re-fixed). Embryos were washed with PBT and pre-hybridised in hybridisation mix (50% formamide, 5X SSC (0.75 M NaCl and 75 mM triNa citrate) at pH 5.0, 500 μg/ml yeast RNA, 50 μg/ml heparin, 0.1% Tween20) for 2 hours at 65°C. The mixture was replaced by fresh hybridisation mix containing DIG-labelled or Fluorescein-labelled RNA probe at the appropriate dilution and incubated at 65°C overnight. The post hybridisation washes were carried out at 65°C. The excess probe was removed by sequential washing in 25% formamide in 2X SSC, 2X SSC, 0.2X SSC at 65°C and finally in PBT at RT. Embryos were incubated in maleic acid buffer (0.1 M Maleic acid pH 7.5 and 0.15 M NaCl) containing 2% Blocking Reagent (Roche; MAB) at RT for a minimum of 1 hour. Staining was carried out by replacing the MAB solution with the 1:5000 MAB diluted alkaline phosphatise (AP) conjugated anti-DIG antibody (Roche) or 1:4000 MAB diluted AP conjugated anti-Fluorescein antibody (Roche) at 4°C overnight. The embryos were washed with PBT and then detection buffer (0.1 M Tris-HCl pH 9.5, 0.1 M NaCl and 50 mM MgCl_2 _and 0.1% Tween20) at RT. The AP substrates used for colour development were BM purple (Roche), Fast Red tablets (Roche) and NBT/BCIP (Roche). The colour reaction was terminated by washing in PBT and embryos were re-fixed in 4% PFA/PBT for 2 hours at RT.

### Immunocytochemistry

Wholemount immunocytochemistry (WICC) was conducted as described previously [22]. Briefly, embryos were fixed overnight at 4°C and all subsequent steps were performed at RT. Embryos were incubated in blocking buffer prior to incubation in primary antibody in 1% goat serum in phosphate buffered saline supplemented with 0.8% triton X100 (PBST) at room temperature. The primary antibodies used were 34 C, F59 and MF20 at dilutions of 1:250, 1:10 and 1:100, respectively. Embryos were rinsed in phosphate buffer with 0.8% triton (PBT) and incubated in a 1:1000 dilution of goat anti-mouse IgG Cy™-5 linked secondary antibody (Amersham) made up in 1% goat serum in PBT overnight. Embryos were rinsed in PBT and stored in 50% glycerol/50% PBS.

For the double immuno-labelling (WISH/WICC), the WISH labelled embryo was initially developed using reagents as described above, followed by antibody labelling using a 1:10 dilution of F59 and a 1:5000 dilution of goat anti-mouse horseradish peroxidase (HRP) conjugated secondary antibody (Merck) carried out as described in the Zebrafish Book [23]. Colour development by HRP was activated by the addition of 0.01% v/v DAB and 6% v/v H_2_O_2_. Reaction was terminated by the addition of PBS and fixed overnight in 4% PFA at 4°C. For double-fluorescent labelling, the WISH labelled embryo was initially developed using Fast Red, followed by antibody labelling as described above.

Embryos were mounted in 100% glycerol and for flat mounting the yolk of the stained embryo was initially removed. Brightfield and fluorescent single or Z-stack images were collected using a X10, X20, X63 oil immersion or X100 water immersion objectives on a Zeiss LSM 510 microscope and LSM Image Examiner software. LSM Image Browser (Version 4,2,0,121) software was used for image post-processing such as preparing three dimensional (3D) projected cross-sections from the acquired Z-stacks of images. For the observation of the Cy™5, Fast Red and Alexa Fluor^® ^(Alexa Fluor^® ^488 nm) staining, argon lasers 633 nm, 543 nm and 488 nm lines and LP 650, LP 560 and BP 505-550 emission filter(s) were used, respectively.

## Results

### The relationship between ryanodine receptor genes in zebrafish and those of other vertebrates

We conducted a tBLASTn search of the zebrafish genomic database (Zv7 Ensembl), using the protein sequences for human RyR1, RyR2 and RyR3 as queries, and detected four zebrafish RyRs homologues: *ryr1a*, *ryr1b*, *ryr2b *and *ryr3 *(as summarised in Table 1). *ryr2a *was identified from scaffold sequences by blasting the human RyR2 sequence in the updated NCBI database. Despite the fact that the sequence obtained from *ryr2a *is not a completed version based on the available information in current databases, the five genomic contigs (NA_1034, NA_1216, NA_3083, NA_1397 and NA_1713) identified to date show high similarity to other vertebrate RyR orthologues. Zebrafish *ryr *gene sequences display significant homology to their respective human isoforms (Table 1).

**Figure 2:**
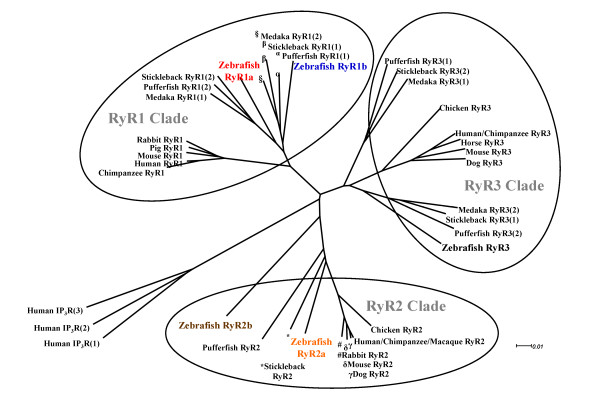
**Phylogenetic tree of the vertebrate RyR gene family**. The evolutionary relationship between the zebrafish *ryr *orthologues and cloned full-length *ryr *genes of other vertebrate species was determined. RyR protein sequences were aligned using the T-coffee multiple sequence alignment. The phylogenetic tree was generated using the equal angled method available on the SplitTree4 software. The branch length of the tree is proportional to evolutionary distance. The family of human inositol 1,4,5-trisphosphate receptors (IP_3_R) was used as an out-group. Three monophyletic clades corresponding to (1) RyR1, (2) RyR2 and (3) RyR3 gene subfamilies were identified. A full description of the sequences used can be found in Table 2. through to adulthood.

The evolutionary relationship of vertebrate RyR genes, including the five zebrafish sequences was examined (Figure 2). Three major monophyletic clades were identified that correspond to RyR1, RyR2 and RyR3 subfamilies. We found that the zebrafish RyR1a, RyR1b, RyR2a, RyR2b and RyR3 all clustered with their respective gene families into the three monophyletic clades [24]. The zebrafish RyR1a and RyR1b cluster to their respective subgroups with those of the other teleost species, *Gasterosteus aculeatus *(stickleback), *Oryzias latipes *(medaka) and *Takifugu rubripes *(pufferfish). We identified two *ryr2 *genes in zebrafish, which currently appears to be the only species in which this gene duplication event can be observed. The zebrafish *ryr2a *gene appears to be more closely related to other teleosts (i.e. stickleback and pufferfish) whereas *ryr2b *gene is more diverged. Although most teleosts appear to have two *ryr3 *genes, we confirmed previous studies which report a single zebrafish *ryr3 *gene [11,13,24].

### Zebrafish RyRs contain many of the conserved structural domains with similarities to other vertebrates

The molecular structure of the RyR protein has been explored extensively, partly in the drive to understand its regulation. Each monomeric RyR protein is approximately 5,000 amino acids in length with a molecular mass of 565 kDa. The receptor has a large N-terminal cytoplasmic domain containing many regulatory binding sites (as reviewed in [25,26]) that modulate the gating of the channel pore located in the C-terminus. The N-terminus cytoplamic domain of RyR interacts with a host of regulatory proteins, such as calstabin and calmodulin. Physiological modulators of RyR function include ATP, Ca^2+^, Mg^2+^, cyclic ADP ribose, posttranslational modifications (e.g. phosphorylation, oxidation) and pharmacological substances (e.g. ryanodine, caffeine) [27].

We conducted an analysis to explore whether the zebrafish RyR protein sequences contain characterised conserved domains that may contribute to receptor regulation and their comparison to their human counterparts was analysed (Figure 1a). MIR (Mannosyltransferase, Inositol 1,4,5-trisphophate receptor (IP_3_R) and RyR [pfam02815]) and RIH (RyR and IP_3_R Homology [pfam01365]) domains were identified within the N-terminal of all five zebrafish RyRs. The MIR and RIH domains are common to all the members of the intracellular Ca^2+^-release channel super family [28,29]. The MIR domain has been suggested to have a ligand transferase function and the RIH domain may form a binding site for IP_3_; however, very little is known regarding their role in receptor regulation to date [29]. All of the zebrafish RyRs contain three SPRY domains (SPla and the RyR [pfam00622]), which have been proposed to interact with voltage gated channels [30,31]. Each zebrafish RyR also contains four copies of the RyR domains (RyR repeated domain [pfam02026]), a sequence unique to these channels [29]. Furthermore, zebrafish RyRs contain the eukaryotic RIH associated (RyR and IP_3_R Homology associated [pfam08454]) domain, which currently has no known function. EF-hand motifs may have a functional significance in activation of the channel by Ca^2+ ^themselves. Putative Ca^2+ ^binding sequences, EF1 and EF2 [32], have been identified in the receptor and are thought to be the major Ca^2+ ^regulatory sites. We identified similar putative EF-hand motifs towards the C-terminal of the receptor and these appear to be conserved in all zebrafish and human isoforms. The RyR pore determines the conductance and ion selectivity of the channel; however, the structure of the TM and pore forming region is still unresolved. There are eight proposed TM sequences, of which the last six are suggested to form the Ca^2+ ^release channel [33]. According to the topological model by Du and colleagues six to eight TM sequences (i.e. M4a/M4b, M5, M6, M7a/M7b, M8 and M10) were identified, with the M9 (pore segment) inserted between the M8 and M10 TM segments. This M9 region is proposed to act as the selectivity filter allowing Ca^2+ ^to transverse the membrane [34]. All five zebrafish RyRs contain RR TM 4-6 domains (RyR TM 4-6 [pfam06459]). Comparison of sequence in the core pore-forming region demonstrate that all five zebrafish RyR sequences are highly conserved with other vertebrate species, although it should be noted that there are subtle changes at the single amino acid level (Figure 1b).

### Zebrafish *ryr *mRNA expression can be detected from the earliest stages of development through to adulthood

**Figure 3:**
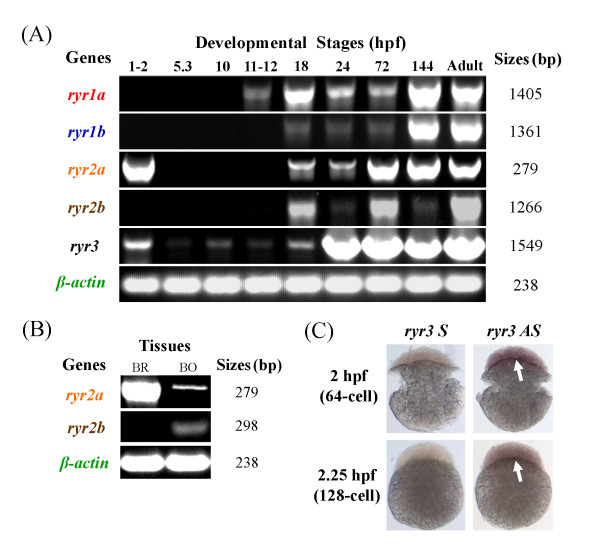
**Zebrafish *ryr *mRNA can be detected from very early developmental stages through to adulthood**. **(a) **Temporal expression pattern of zebrafish *ryrs *using end-point PCR analysis and isoform specific primers. Total RNA was isolated from different developmental stages, as indicated, and reverse transcribed. 1.5 μg of cDNA was synthesised and isoform specific primers for ryr1a, ryr1b, ryr2a, ryr2b and ryr3 used. Numbers in the right panel indicate the molecular mass of PCR products. *β-actin *was used as an internal control. The figure shows a representative result of replicates from at least three experiments. **(b) **Expression of zebrafish ryr2a and ryr2b in the brain (BR) and body (BO) of a 3 months old adult zebrafish. *β-actin *was used as an internal control. Numbers in the right panel indicate the molecular mass of RT-PCR products. **(c) **Maternal expression of ryr3 mRNA was observed as staining (arrows) within the dividing cells of 2-2.25 hpf embryos (64 and 128-cell stage). The specificity of the signal is compared to the ryr3 sense probes.

We examined the temporal expression of the *ryr *genes during nine key stages of zebrafish development using semi-quantitative end-point PCR (Figure 3a). Expression of *ryr1a *was detected from 11-12 hpf (3- to 6-somite stage) through to adulthood, whereas *ryr1b *expression was evident from 18 hpf (18-somite stage) onwards. Results also showed that levels of mRNA expression for both *ryr1a *and *ryr1b *were at the highest in 144 hpf and adult stages. The *ryr2a *gene showed strong maternal expression at 1-2 hpf (4- to 128-cell stage) and zygotic expression was observed from 18 hpf through to adulthood. Expression of *ryr2b *mRNA was detected from 18 hpf through to adulthood with relatively weak expression observed at 24 hpf and 144 hpf stages. Finally, *ryr3 *maternal expression was observed at 1-2 hpf, followed by weak zygotic mRNA expression from 5.3 hpf (50% epiboly stage) through to 18 hpf (18-somite stage), after which the levels became much stronger ***ryr1a*, *ryr1b *and *ryr3 *are expressed exclusively in skeletal muscle during segmentation period**

**Figure 4:**
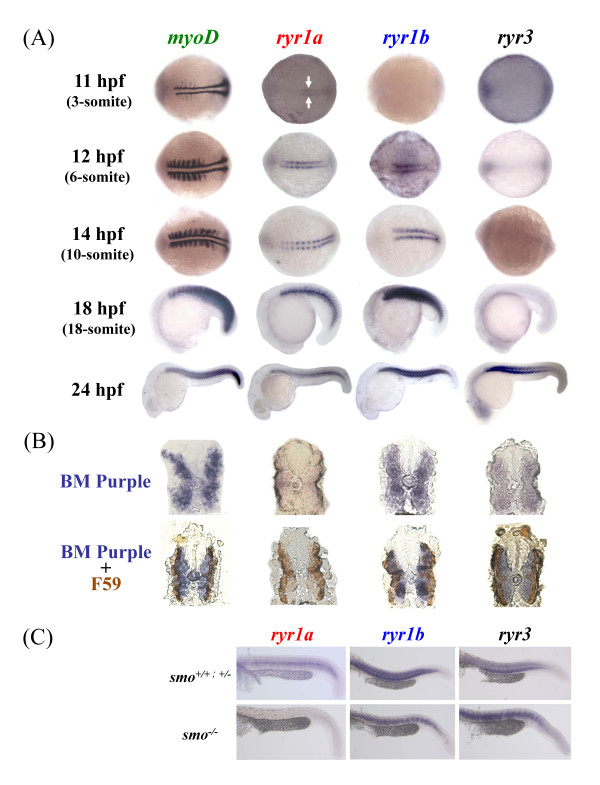
**Zebrafish ryr1a, ryr1b and ryr3 mRNA is localised to embryonic skeletal muscle**. **(a) **Expression of zebrafish *myoD*, ryr1a, ryr1b and ryr3 was examined using whole mount in situ hybridisation. Expression of ryr1a mRNA is detectable in the adaxial cells of 11 hpf embryos (3-somite, arrows), whereas ryr1b mRNA expression is present in cells adjacent to the notochord from 12 hpf (6-somite stage) embryos. Expression of ryr3 mRNA was only evident at 24 hpf, with the strongest staining observed in the anterior somites. The embryos at 11 and 12 hpf are orientated so that the anterior is to the left. **(b) **Cross-sections showing *myoD*, ryr1a, ryr1b and ryr3 in-situ mRNA hybridisation (above) and double immunostained with the F59 antibody/HRP labeling (below) in 24 hpf embryos. (**c**) ryr1a, ryr1b and ryr3 mRNA expression in wildtype (*smo^+/+^*), heterozygote (*smo^+/−^*) and homozygous (*smo^−/−^*) mutants at 24 hpf. There is no ryr1a mRNA expression in the *smo^−/− ^*mutant, compared to wildtype and heterozygous embryos at 24 hpf.

We examined the spatial and temporal distribution pattern of the zebrafish *ryr *mRNA in the developing embryo from 12 to 24 hpf of development using WISH. The expression of *ryr1a*, *ryr1b *and *ryr3 *in the developing somites was compared to the myogenic determinant (*myoD*), one of the earliest markers of myogenic commitment with a key role in regulating muscle differentiation (Figure 4a) [19].

We observed low levels of *ryr1a *expression in the adaxial cells located on either side of the notochord beginning at 11 hpf (Figure 4a). Our findings demonstrate that in slow muscle *ryr1a *appears at 11 hpf, 1 to 2 hpf prior to fast muscle *ryr1b *mRNA expression. After the lateral migration of the slow muscle precursors is completed, the expression of *ryr1a *mRNA is clearly visible in the superficial layer of slow muscle (Figure 4b). There is conflicting evidence that by 24 hpf *ryr1a *mRNA is confined either to slow muscle fibres [13], or can be detected at low levels throughout the somite[14]. In this study we found that *ryr1a *mRNA co-localises with a known marker of slow muscle fibres at 24 hpf (Figure 4b). Furthermore, *ryr1a *mRNA staining was not detected in the somites of smoothened (*smo−/−*) embryos, a mutant line which lacks slow muscle and the muscle pioneers (Figure 4c). Thus, we conclude that *ryr1a *mRNA is expressed exclusively in embryonic slow muscle.

We observed low levels of *ryr1b *expression in adaxial cells adjacent to the notochord beginning at 12-13 hpf (Figure 4a). At 18 and 24 hpf, strong *ryr1b *expression was detected throughout the somites in a pattern analogous to that of *myoD*, suggestive of its presence in both fast and slow muscle. Currently there is conflicting reports suggesting that *ryr1b *expression is confined either to the fast muscle [13] or appears in both fast and slow muscle [35]. In the later study the observation that *ryr1b *mRNA expression is found in both muscle types was attributed to an artefact arising as a consequence of contaminated tissue [35]. In order to clarify our findings we performed double-fluorescent labeling experiments, to observe *ryr1b *expression in the presence of a slow muscle marker. In contrast to previous work which used a *ryr1b in situ *probe that targeted the relatively conserved pore forming region at the C-terminus we generated a probe that recognised a more variable region at the N-terminus of the receptor [13]. Our data revealed that *ryr1b *mRNA is localised to both fast and slow developing skeletal muscle (Figure 4b, Additional file 1: Figure S1).

Maternal expression of *ryr3 *in the dividing cells of 2 to 2.25 hpf (64- and 128-cell) embryos was confirmed by wholemount *in situ *hybridisation (WISH) (Figure 3b); however, the low level expression of *ryr3 *from 5.3 to 18 hpf detected in the PCR analysis was not confirmed by WISH (data not shown). In this study *ryr3 *mRNA expression was detected in the skeletal muscle from 24 hpf using WISH (Figure 4a,b), with stronger expression in the anterior compared to developing posterior somites (Figure 4a). There is conflicting evidence that *ryr3 *mRNA in zebrafish is expressed exclusively in slow and fast skeletal muscle from 14 hpf [11] or in many tissues including the CNS during the somitogenesis [14,36]. The discrepancies in the expression patterns of *ryr3 *mRNA may be explained in part by the target sequence used for probe synthesis in previous studies. The *ryr3 *clone (019-D04-2) used previously targeted the conserved pore-forming region at the C-terminus of the receptor which shares high similarity to both the *ryr1a *and *ryr1b *sequence, this raises the possibility that cross hybridisation with *ryr1 *isoforms occurred [11]. In the current work the *ryr3 *probe was designed to a more divergent region located in the N-terminus of the receptor. In this study *ryr3 *mRNA expression was not detected in the Kuppfer's vesicles, adaxial cells at 12 hpf or in any region of the CNS at 24 hpf (data not shown). Double staining experiments revealed that *ryr3 *mRNA expression was confined to the fast skeletal muscle at 24 hpf (Figure 4b, Additional file 1: Figures S1 and Additional file 2: Figure S2). Thus we conclude that *ryr3 *mRNA is expressed exclusively in the fast skeletal muscle of embryos at 24 hpf.

### *ryr2a *and *ryr2b *are expressed in the developing zebrafish CNS and heart, respectively

**Figure 5:**
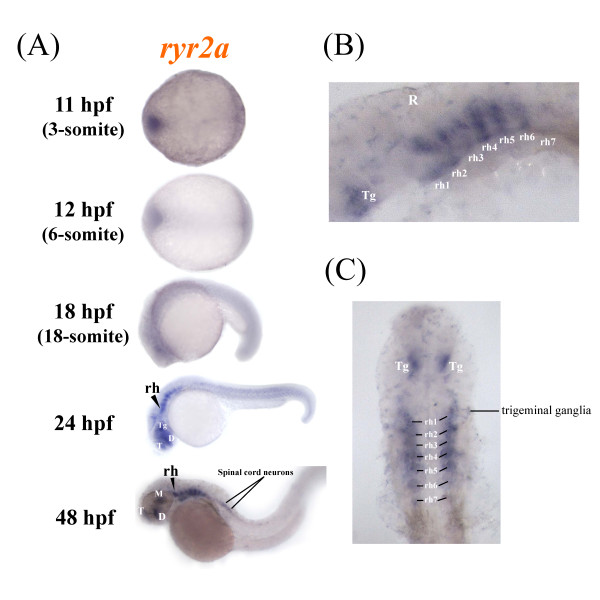
**Zebrafish ryr2a is expressed in the central nervous system**. The spatial distribution of ryr2a mRNA in the zebrafish embryo was examined using whole mount in situ hybridisation. **(a) **Specific staining was observed initially in localised regions of the brain, specifically the telencephalon (T), diencephalon (D), mesencephalon (M), tegmentum (Tg) and rhomobomeres (rh) of whole mount embryos at 24 and 48 hpf. **(b) **Lateral and **(c) **dorsal views (eyes removed) of the brain at 48 hpf revealed that ryr2a expression was present in each of the seven rhombomere segments, the tegmentum, trigeminal ganglia and anterior portion of the spinal cord. Embryos are orientated so that anterior is to the left.

Expression of *ryr2a *mRNA was detected initially in the CNS of zebrafish embryos starting at 24 hpf (Figure 5a). At 24 and 48 hpf, *ryr2a *mRNA expression can be observed at several sites in the developing forebrain (telencephalon and diencephalon), midbrain (mesencephalon) and hindbrain (rhombencephalon) of the zebrafish embryo (Figure 5a). A lateral view shows *ryr2a *mRNA expression in all seven hindbrain rhombomeres, r1-r7 by 48 hpf (Figure 5b). The dorsal view clearly revealed two bilateral patches of *ryr2a *expression lying dorsal to the eyes which may correspond to the developing tegmental region (Figure 5c). Low level staining was also observed in the anterior portion of the spinal cord at 48 hpf (Figure 5a). Analysis of *ryr2a *expression by PCR showed that 3 month old zebrafish display a strong *ryr2b *mRNA expression in the brain compared to the body (Figure 3b). However, the significant maternal expression of *ryr2a *mRNA detected by PCR (Figure 3a) was not detected by WISH (data not shown). We conclude that zygotic *ryr2a *expression is expressed exclusively in the developing CNS of zebrafish embryos.

**Figure 6:**
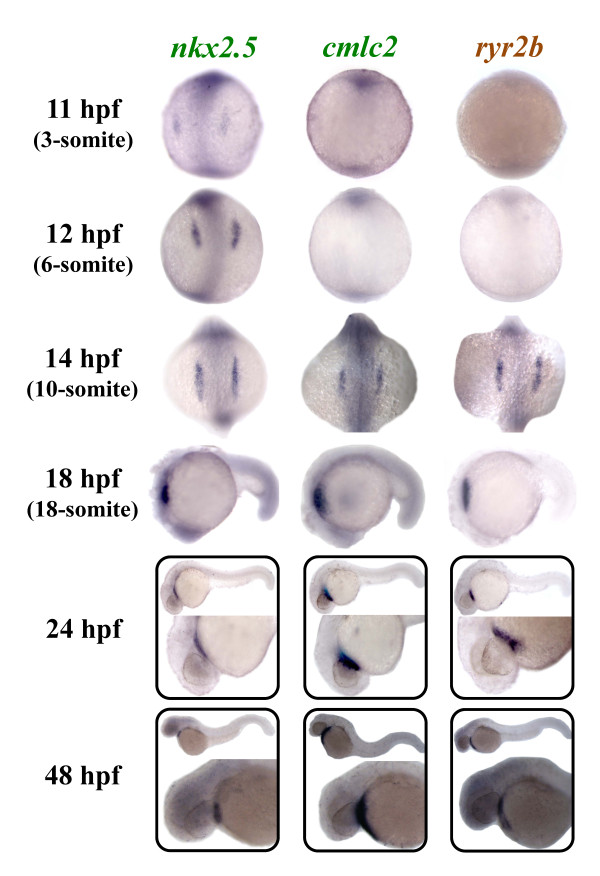
**Zebrafish ryr2b is expressed in the differentiating cardiac muscle**. Expression of zebrafish nkx2.5, *cmlc2*, and *ryr2b *was examined using whole mount in situ hybridisation. Expression of *nkx2.5 *was observed in myocardial precursors from 11 hpf (3-somite stage) onwards, whereas *cmlc2 *and ryr2b were expressed in differentiating cardiac tissue from 14 hpf (10-somite stage) onwards. 11, 12 and 14 hpf embryos are orientated so that the anterior is to the top, whereas in 18, 24 and 48 hpf embryos anterior is to the left.

**Figure 7:**
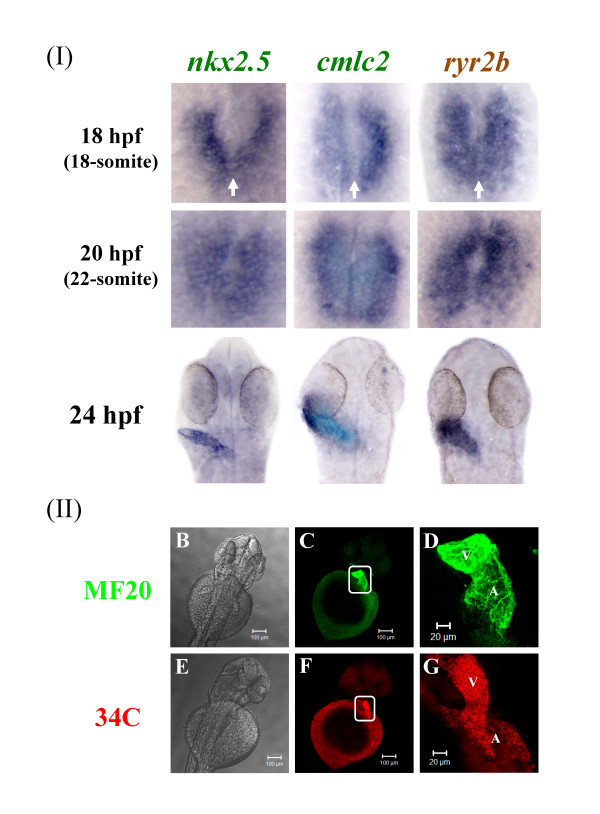
**Zebrafish ryr2b mRNA is expressed during early heart formation and the RyR protein throughout the heart at later stages**. **(a) **Expression of *nkx2.5*, *cmlc2*, and ryr2b in 18 hpf (18-somite stage) was observed in the bilateral cardiac primordial cells. At this stage the bilateral cardiac primordial cells make contact and begin to fuse (arrows). The posterior portions fuse initially followed by anterior portions to create a central lumen and cardiac cone by 20 hpf (22-somite stage). A linear heart tube has formed by 24 hpf and ryr2b and *cmlc2^+ ^*cells are expressed throughout the heart at this stage. 18- and 22-somite stage embryos, in which the tail was removed but the yolk sac left intact, were orientated so that anterior is to the top. Dorsal view of flat mounted embryos at 24 hpf. **(b&e) **Brightfield images of the anterior portion of the zebrafish embryo with the yolk sac still intact and dorsal side uppermost. Embryos were fixed and stained at 48 hpf to reveal either myosin **(c-d) **or RyRs **(f-g)**, the images within the white boxes are shown in greater detail **(d & g)**. By 48 hpf RyRs are expressed throughout the two chambers, atrium (A) and ventricle (V) of the heart. Images were taken using X10 **(b,c & e,f) **and X20 **(d & g) **objectives.

The expression of *ryr2b *was compared to two well-characterised cardiac markers, *nkx2.5 *and *cmlc2 *[18,20,37] (Figure 6). Expression of *nkx2.5*, a homeodomain transcription factor, is the earliest known marker of zebrafish precardiac mesoderm [20,37]. Cardiac myosin light chain 2 (*cmlc2*), another marker of myocardial precursor cells, is expressed throughout heart fusion and early heart tube assembly [18]. We show that *nkx2.5 *but not *cmlc2 *and *ryr2b *expression can be observed in the heart progenitor cells at 11 hpf; however, by 14 hpf the bilateral expression of *cmlc2 *and *ryr2b *was apparent in the precardiac mesoderm. As reported previously [18], *nkx2.5 *expression extends beyond the anterior tip of the notochord and the most posterior *nkx2.5 *cells (*nkx2.5^+^*) do not express *cmlc2 (cmlc2^-^). ryr2b *expression appears to be expressed in the region corresponding to *cmlc2^+ ^*consistent with its appearance in cells that will contribute to the myocardium. By 18 hpf the bilateral cardiac primordial cells expressing *cmlc2 *and *ryr2b *bend towards each other, make contact and begin to fuse (Figure 7a). The posterior portions fuse initially followed by anterior portions to create a central lumen and cardiac cone by 20 hpf. At 24 hpf, the heart has formed a linear tube with atrial and ventricular precursor cells and *ryr2b *expression appears to mirror that of *cmlc2^+^*cells, suggestive of its expression throughout the heart at this stage. Using WICC, we observed RyR protein in the atrium and ventricle of the zebrafish heart at 48 hpf (Figure 7b).

## Discussion

### Structure of the zebrafish *ryr *genes and their products: comparison to other species

Previous work identified a total of 14 genomic contigs for *ryr *from the zebrafish genome assembly [13]. Classification of RyR sequences by radiation hybrid mapping suggested that there are at least five different zebrafish genes: *ryr1a*, *ryr1b*, *ryr2a*, *ryr2b *and *ryr3 *[13]. Our study has confirmed the sequences of the five zebrafish *ryr *genes, although currently the *ryr2a *gene annotation is still incomplete. Zebrafish, like other teleosts, have undergone a gene duplication event and appear to have retained two distinct copies of *ryr1 *and *ryr2*. The teleost *ryr1 *genes are differentially expressed in the skeletal muscle tissue and the receptors have distinctive Ca^2+ ^binding sensitivities, suggesting that the genes have evolved to perform different physiological functions [13,38]. Here we report that the zebrafish genome contains two copies of the *ryr2 *gene, currently the only teleost in which this gene duplication can be observed. Our study has revealed that expression of *ryr2a *and *ryr2b *is confined to the embryonic nervous and cardiac tissue respectively. The differential expression of the *ryr2 *supports the idea that the gene products have different roles within these tissues. The reported sub-functionalisation of the *ryr2 *in the zebrafish embryo in this study will facilitate the study of this receptor in the development of nervous and cardiac tissue.

Our work has revealed that the primary sequence of the zebrafish RyRs contains many of the conserved domains associated with the regulation and function of this intracellular ion channel in other species, mostly notably humans. The primary sequence of the Ca^2+^-conducting pore domain was found to be extensively conserved between zebrafish and other species examined. Our study of RyR primary structure supports previous work to show that the biophysical properties and pharmacological regulation of the zebrafish RyR1 is similar to its mammalian homolog [39]. Taken together this data reveals that the zebrafish RyR functions in a similar manner to those found in mammals, this information is of significant relevance to work using the zebrafish as a model for human disease. However, differences in the predicted primary sequence of the zebrafish ryanodine receptor proteins and those of other species were also recorded. There are reports species-specific differences in the Ca^2+ ^regulation and single channel conductance of the RyR1 channel [39]. Further comparative analysis of the RyR family will provide a better insight into the physiological functions of the receptor at a tissue or whole organism level.

### The expression of *ryr *genes during early development, up to and including axis formation

Calcium signalling is required throughout development; however the signal pathways have not been well defined. The prevailing view is that IP_3_R-driven Ca^2+ ^signals have a major role in axis formation prior to organogenesis whereas RyR-induced Ca^2+ ^signals are necessary for the later aspects of tissue specific differentiation (e.g. muscle formation) [3-5,7,9,40]. Our current study revealed strong maternal expression of *ryr2b *and *ryr3 *genes during cleavage, with low levels of *ryr3 *expression detectable throughout the blastula and gastrula periods. The significance of early *ryr *mRNA expression remains to be determined; however, it raises the possibility that RyR-generated Ca^2+ ^signals act in development prior to 10 hpf. The characterisation of expression in zebrafish establishes a basis for future experimental work aimed at determining the action of RyR-induced Ca^2+ ^signalling events in early embryonic patterning.

### The expression of *ryr *genes in developing skeletal muscle

Several studies have implicated RyR function in organogenesis, particularly in striated muscle development (as reviewed in [9]). Our study has used both mutant lines and double staining to establish that RyRs are differentially expressed in slow (*ryr1a*), fast (*ryr3*) and both types (*ryr1b*) of developing skeletal muscle during the segmentation period. In E-C coupling within mammalian skeletal muscle, RyR1 is directly coupled to a voltage gated-Ca^2+ ^channel (VOC) on the sarcolemma. Activation of VOCs via membrane depolarisation then triggers the opening of the ryanodine receptor (RyR1) and release of Ca^2+ ^from the sarcoplasmic reticulum (SR) stores. Zebrafish have non-Ca^2+^-conducting voltage-gated Ca channels that have evolved solely as voltage sensors to trigger opening of the RyR [35]. The central subunit of the VOC (Ca_v1.1α1s_) acts as the pore, selectivity filter and voltage sensor. In zebrafish two Ca_v1.1α1s _genes (*zf-α1s-*a and *zf-α1s-b*) have been identified. The Ca_v1.1α1s _gene products are proposed to interact in a tissue specific manner with the *ryr1 *genes; that is *zf-α1s-a *and *ryr1a *are expressed in slow muscle whilst *zf-α1s-b *and *ryr1b *are confined to fast muscle. However, our data suggest that the situation is not quite as clear cut as first proposed because *ryr1b *is not expressed exclusively in fast muscle but is also located in slow muscle. In addition, Ca^2+ ^release can also be regulated by the *ryr3 *gene product which is also located in the fast skeletal muscle. RyR3 is proposed to act as an uncoupled calcium-induced calcium release (CICR) channel to propagate the Ca^2+ ^signal [41]. Therefore we propose that in the developing fast muscle *zf-α1s-b *and *ryr1b *act together to generate an increase in [Ca^2+^]_i_, with the *ryr3 *gene product acting to amplify the signal. Our data in the embryonic fast muscle showed that *ryr1b *expression occurs prior to *ryr3 *and suggests that the RyR-generated [Ca^2+^]_i _increase occurs initially via *ryr1b *with the proposed amplification step via *ryr3 *developing subsequently. In zebrafish the role of the RyR in E-C coupling within the developing slow muscle appears more complex. Our data reveals that both *ryr1a *and *ryr1b *are expressed in the developing slow muscle, but *ryr3 *is not. This presents the possibility that *zf-α1s-a *could couple to both *ryr1a *and *ryr1b *and raises the issue of whether amplification of the Ca^2+ ^signals occurs in this tissue and, if so, how is this achieved. Clearly there is still much to understand about the maturation of depolarization-induced Ca^2+ ^signaling and its role during skeletal muscle differentiation *in vivo*.

### The expression of *ryr *genes in the developing nervous and cardiovascular systems

Our data has revealed that there are two *ryr2 *genes, *ryr2a *and *ryr2b*, which are exclusively expressed in either the developing nervous system or cardiac tissue, respectively. Studies in mammals revealed that RyR are expressed in the developing brain and that RyR-mediated Ca^2+ ^signals may have a role in neuronal differentiation and neurite outgrowth [42-44]. All three *ryr *genes are expressed within the embryonic mouse brain; however, from postnatal day 7 onwards *ryr2 *becomes in the major isoform [45]. The postnatal changes in RyR expression in mouse brain correlate with a period of neuronal differentiation and may therefore be important in establishing [Ca^2+^]_i _homeostasis in maturing neurons. In zebrafish *ryr2a *expression is localized to specific regions of the developing brains. In these regions *ryr2a *is likely to regulate neuronal Ca^2+ ^signaling and therefore play a role in CNS development.

In mature cardiac muscle, Ca^2+ ^signals are generated by CICR via the activation of VOCs and the cardiac RyR (RyR2). Knockout mice which do not express *ryr2 *initially display spontaneous rhythmic contractions of the heart at embryonic day 9 (E9) but no heart beat by day 10 (E10) [4]. Furthermore, RyR-mediated Ca^2+ ^release does not play a significant role in the [Ca^2+^]_i _changes observed within the heart of new born rats [46]. Thus in mammals it appears that the RyR2 does not contribute to the onset of contractile activity at very early embryonic stages, but is important for the subsequent maturation and development of the heart *in vivo*. The zebrafish cardiac *ryr *gene (*ryr2b) *is expressed exclusively in the developing heart tissue (precardiac mesoderm) from 14 hpf, 8 hours prior to the onset of cardiac contraction at 22 hpf, and may well contribute to early cardiac development. Investigation of *ryr2 *function during mammalian development is complicated by the fact that a single *ryr2 *gene is expressed in several tissues [1]. The sub-functionalisation of the *ryr2a *and *ryr2b *genes in the zebrafish embryo provides an excellent system to study individual receptor function in neuronal and cardiac tissues during vertebrate development.

## Conclusions

This study has provided a comprehensive overview of the spatial and temporal expression of the *ryr *gene family in developing zebrafish embryos. This family of Ca^2+^-release channels are expressed predominantly in developing skeletal, cardiac and neuronal tissue, supportive of the view that RyRs function is relevant to later development events, such as tissue differentiation. In addition, the study has also revealed that maternal *ryr *mRNA is present in the very early embryo, suggestive of a function for this receptor prior to organogenesis. Ryanodine receptors have been implicated in human disease and the zebrafish is an important vertebrate developmental model which will facilitate work in this area. Future work will explore the function of RyR-regulated Ca^2+ ^signal pathways during zebrafish embryogenesis.

## Competing interests

The authors declare that they have no competing interests.

## Authors' contributions

H.H.T.W performed all the experimental work and contributed to the writing of the manuscript. C.B. supervised and provided input on the design of the experiments. R.A. designed and supervised the experiments and wrote the manuscript. All authors read and approved the final manuscript.

## Supplementary Material

Additional file 1 **Figure S1**. **ryr1a, ryr1b and ryr3 mRNA is differentially expressed in the developing skeletal muscle**. Double labelling in wholemount embryos at 24 hpf was performed using probes to *myhz1*, ryr1a, ryr1b and ryr3 and Fast Red as a substrate (red) followed by immunostaining using the F59 antibody and a fluorescent secondary (green). Images show Z-stacks of whole-mount double-labelled embryos (top row) or sections (bottom row) from dissected embryos. Cross-sectional images revealed that ryr1a co-localised exclusively with slow muscle staining, whereas *myhz1*, a marker of fast muscle, was not expressed in the slow muscle. Furthermore ryr1b expression could be observed throughout both muscle types whereas *ryr3 *did not co-localise with the slow muscle staining and appeared to be expressed exclusively in the fast muscle. Scale bars = 20 μm, unless otherwise indicated. Click here for file

Additional file 2 Figure S2. **ryr3 mRNA expression is confined to the fast muscle fibres throughout the myotome at 24 hpf**. Double fluorescent cross-sections (1-3) were prepared by labelling a 24 hpf zebrafish embryo with a fluorescence substrate (i.e. FastRed; red) for ryr3 in situ hybridisation and the F59 antibody (green) for immunostaining. The position of cross-sections 1-3 are illustrated in the 24 hpf ryr3 WISH labelled embryo (top left) which has been stained with BM purple and laterally orientated with anterior to the left. Scale bars = 20 μm, unless otherwise indicated. Click here for file

## References

[B1] Sutko JL Airey JA Ryanodine receptor Ca2+ release channels: does diversity in form equal diversity in function? Physiol Rev 1996 76 1027 71 8874493 10.1152/physrev.1996.76.4.1027

[B2] Betzenhauser MJ Marks AR Ryanodine receptor channelopathies Pflugers Arch 460 467 80 10.1007/s00424-010-0794-4 PMC2885589 20179962

[B3] Takeshima H Iino M Takekura H Nishi M Kuno J Minowa O Takano H Noda T Excitation-contraction uncoupling and muscular degeneration in mice lacking functional skeletal muscle ryanodine-receptor gene Nature 1994 369 556 9 10.1038/369556a0 7515481

[B4] Takeshima H Komazaki S Hirose K Nishi M Noda T Iino M Embryonic lethality and abnormal cardiac myocytes in mice lacking ryanodine receptor type 2 EMBO J 1998 17 3309 16 10.1093/emboj/17.12.3309 9628868 PMC1170669

[B5] Takeshima H Ikemoto T Nishi M Nishiyama N Shimuta M Sugitani Y Kuno J Saito I Saito H Endo M Generation and characterization of mutant mice lacking ryanodine receptor type 3 J Biol Chem 1996 271 19649 52 10.1074/jbc.271.33.19649 8702664

[B6] Bertocchini F Ovitt CE Conti A Barone V Scholer HR Bottinelli R Reggiani C Sorrentino V Requirement for the ryanodine receptor type 3 for efficient contraction in neonatal skeletal muscles EMBO Journal 1997 16 6956 6963 10.1093/emboj/16.23.6956 9384575 PMC1170299

[B7] Rosemblit N Moschella MC Ondriasa E Gutstein DE Ondrias K Marks AR Intracellular calcium release channel expression during embryogenesis Dev Biol 1999 206 163 77 10.1006/dbio.1998.9120 9986730

[B8] SE Webb AL Miller Calcium signalling during embryonic development Nat Rev Mol Cell Biol 2003 4 539 51 10.1038/nrm1149 12838337

[B9] Slusarski DC Pelegri F Calcium signaling in vertebrate embryonic patterning and morphogenesis Dev Biol 2007 307 1 13 10.1016/j.ydbio.2007.04.043 17531967 PMC2729314

[B10] Ashworth R Approaches to measuring calcium in zebrafish: focus on neuronal development Cell Calcium 2004 35 393 402 10.1016/j.ceca.2004.01.002 15003849

[B11] Brennan C Mangoli M Dyer CE Ashworth R Acetylcholine and calcium signalling regulates muscle fibre formation in the zebrafish embryo J Cell Sci 2005 118 5181 90 10.1242/jcs.02625 16249237

[B12] Chi NC Shaw RM Jungblut B Huisken J Ferrer T Arnaout R Scott I Beis D Xiao T Baier H Genetic and physiologic dissection of the vertebrate cardiac conduction system PLoS Biol 2008 6 e109 10.1371/journal.pbio.0060109 18479184 PMC2430899

[B13] Hirata H Watanabe T Hatakeyama J Sprague SM Saint-Amant L Nagashima A Cui WW Zhou W Kuwada JY Zebrafish relatively relaxed mutants have a ryanodine receptor defect, show slow swimming and provide a model of multi-minicore disease Development 2007 134 2771 81 10.1242/dev.004531 17596281

[B14] Jurynec MJ Xia R Mackrill JJ Gunther D Crawford T Flanigan KM Abramson JJ MT Howard DJ Grunwald Selenoprotein N is required for ryanodine receptor calcium release channel activity in human and zebrafish muscle Proc Natl Acad Sci USA 2008 105 12485 90 10.1073/pnas.0806015105 18713863 PMC2527938

[B15] Zimprich F Ashworth R Bolsover SR Real-time measurements of calcium dynamics in neurons developing in situ within zebrafish embryos Pflugers Arch-Eur J Physiol 1998 436 489 493 10.1007/s004240050662 9644235

[B16] Kimmel CB Ballard WW Kimmel SR Ullman B Schilling TF Stages of Embryonic Development of the Zebrafish Dev Dyn 1995 203 253 310 10.1002/aja.1002030302 8589427

[B17] Schulte-Merker S Ho RK Herrmann BG C Nusslein-Volhard The protein product of the zebrafish homologue of the mouse T gene is expressed in nuclei of the germ ring and the notochord of the early embryo Development 1992 116 1021 32 1295726 10.1242/dev.116.4.1021

[B18] Yelon D Horne SA Stainier DY Restricted expression of cardiac myosin genes reveals regulated aspects of heart tube assembly in zebrafish Dev Biol 1999 214 23 37 10.1006/dbio.1999.9406 10491254

[B19] Weinberg ES Allende ML Kelly CS Abdelhamid A Murakami T Andermann P Doerre OG Grunwald DJ Riggleman B Developmental regulation of zebrafish MyoD in wild-type, no tail and spadetail embryos Development 1996 122 271 80 8565839 10.1242/dev.122.1.271

[B20] Lee KH Xu Q Breitbart RE A new tinman-related gene, nkx2.7, anticipates the expression of nkx2.5 and nkx2.3 in zebrafish heart and pharyngeal endoderm Dev Biol 1996 180 722 31 10.1006/dbio.1996.0341 8954740

[B21] Xu Y He J Wang X Lim TM Gong Z Asynchronous activation of 10 muscle-specific protein (MSP) genes during zebrafish somitogenesis Dev Dyn 2000 219 201 15 10.1002/1097-0177(2000)9999:9999<::AID-DVDY1043>3.3.CO;2-9 11002340

[B22] Ashworth R Zimprich F Bolsover SR Buffering intracellular calcium disrupts motoneuron development in intact zebrafish embryos Brain Res Dev Brain Res 2001 129 169 79 11506861 10.1016/s0165-3806(01)00198-5

[B23] Westerfield M The Zebrafish Book: A guide for the laboratory use of zebrafish (Brachydanio rerio) 1995 3 Univ of Oregon Press, Eugene

[B24] Darbandi S Franck JP A comparative study of ryanodine receptor (RyR) gene expression levels in a basal ray-finned fish, bichir (Polypterus ornatipinnis) and the derived euteleost zebrafish (Danio rerio) Comp Biochem Physiol B Biochem Mol Biol 2009 154 443 8 10.1016/j.cbpb.2009.09.003 19755169

[B25] Hamilton SL Ryanodine receptors Cell Calcium 2005 38 253 60 10.1016/j.ceca.2005.06.037 16115682

[B26] Hamilton SL Serysheva I Ryanodine receptor structure: Progress and challenges J Biol Chem 2008 10.1074/jbc.R800054200 PMC3837402 18927076

[B27] Zalk R Lehnart SE Marks AR Modulation of the ryanodine receptor and intracellular calcium Annu Rev Biochem 2007 76 367 85 10.1146/annurev.biochem.76.053105.094237 17506640

[B28] Sorrentino V Barone V Rossi D Intracellular Ca(2+) release channels in evolution Curr Opin Genet Dev 2000 10 662 7 10.1016/S0959-437X(00)00139-8 11088018

[B29] Ponting CP Novel repeats in ryanodine and IP3 receptors and protein O-mannosyltransferases Trends Biochem Sci 2000 25 48 50 10664581 10.1016/s0968-0004(99)01513-3

[B30] Ponting C Schultz J Bork P SPRY domains in ryanodine receptors (Ca(2+)-release channels) Trends Biochem Sci 1997 22 193 4 10.1016/S0968-0004(97)01049-9 9204703

[B31] Cui Y Tae HS Norris NC Karunasekara Y Pouliquin P Board PG Dulhunty AF Casarotto MG A dihydropyridine receptor alpha(1s) loop region critical for skeletal muscle contraction is intrinsically unstructured and binds to a SPRY domain of the type 1 ryanodine receptor Int J Biochem Cell Biol 2008 10.1016/j.biocel.2008.08.004 18761102

[B32] Fessenden JD Feng W Pessah IN Allen PD Mutational analysis of putative calcium binding motifs within the skeletal ryanodine receptor isoform, RyR1 J Biol Chem 2004 279 53028 35 10.1074/jbc.M411136200 15469935

[B33] Du GG MacLennan DH Topology and Transmembrane Organisation of Ryanodine Receptors Ryanodine Receptors: structure, function and dysfunction in clinical disease 2005 Edited by ZHT Wehrens, AR Marks: Springer

[B34] Du GG Sandhu B Khanna VK Guo XH MacLennan DH Topology of the Ca2+ release channel of skeletal muscle sarcoplasmic reticulum (RyR1) Proc Natl Acad Sci USA 2002 99 16725 30 10.1073/pnas.012688999 12486242 PMC139211

[B35] Schredelseker J Shrivastav M Dayal A Grabner M Non-Ca2 + −conducting Ca2+ channels in fish skeletal muscle excitation-contraction coupling Proc Natl Acad Sci USA 2010 107 5658 63 10.1073/pnas.0912153107 20212109 PMC2851825

[B36] Heyer V Lux A Alunni V Degrave A Seiliez I Kirchner J Parkhill JP Thisse C Spatial and temporal expression of the zebrafish genome by large-scale in situ hybridization screening Methods Cell Biol 2004 77 505 19 15602929 10.1016/s0091-679x(04)77027-2

[B37] Chen JN Fishman MC Zebrafish tinman homolog demarcates the heart field and initiates myocardial differentiation Development 1996 122 3809 16 9012502 10.1242/dev.122.12.3809

[B38] Franck JP Morrissette J Keen JE Londraville RL Beamsley M Block BA Cloning and characterization of fiber type-specific ryanodine receptor isoforms in skeletal muscles of fish Am J Physiol 1998 275 C401 15 9688594 10.1152/ajpcell.1998.275.2.C401

[B39] Koulen P Janowitz T Johenning FW Ehrlich BE Characterization of the Calcium-release Channel/Ryanodine Receptor from Zebrafish Skeletal Muscleb Journal of Membrane Biology 2001 183 155 163 10.1007/s00232-001-0063-8 11696857

[B40] Ashworth R Devogelaere B Fabes J Tunwell RE Koh KR De Smedt H Patel S Molecular and functional characterization of inositol trisphosphate receptors during early zebrafish development J Biol Chem 2007 282 13984 93 10.1074/jbc.M700940200 17331947

[B41] Sorrentino V Reggiani C Expression of the ryanodine receptor type 3 in skeletal muscle. A new partner in excitation-contraction coupling? Trends Cardiovasc Med 1999 9 54 61 10.1016/S1050-1738(99)00003-1 10189968

[B42] Faure AV Grunwald D Moutin MJ Hilly M Mauger JP Marty I De M Waard M Albrieux M Developmental expression of the calcium release channels during early neurogenesis of the mouse cerebral cortex Eur J Neurosci 2001 14 1613 22 10.1046/j.0953-816x.2001.01786.x 11860456

[B43] Ooashi N Futatsugi A Yoshihara F Mikoshiba K Kamiguchi H Cell adhesion molecules regulate Ca2 + -mediated steering of growth cones via cyclic AMP and ryanodine receptor type 3 J Cell Biol 2005 170 1159 67 10.1083/jcb.200503157 16172206 PMC2171540

[B44] Lee SM Lee JW Song YS Hwang DY Kim YK Nam SY Kim DJ YW Yun Yoon DY Hong JT Ryanodine receptor-mediated interference of neuronal cell differentiation by presenilin 2 mutation J Neurosci Res 2005 82 542 50 10.1002/jnr.20655 16240390

[B45] Mori F Fukaya M Abe H Wakabayashi K Watanabe M Developmental changes in expression of the three ryanodine receptor mRNAs in the mouse brain Neurosci Lett 2000 285 57 60 10.1016/S0304-3940(00)01046-6 10788707

[B46] Perez CG Copello JA Li Y Karko KL Gomez L Ramos-Franco J Fill M Escobar AL Mejia-Alvarez R Ryanodine receptor function in newborn rat heart Am J Physiol Heart Circ Physiol 2005 288 H2527 40 10.1152/ajpheart.00188.2004 15626694

