# Supplementary figures and images for: Ryanodine receptors, a family of intracellular calcium ion channels, are expressed throughout early vertebrate development
Source: BMC Res Notes. 2011 Dec 14;4:541. doi: 10.1186/1756-0500-4-541 (PMC3262159; doi:10.1186/1756-0500-4-541)

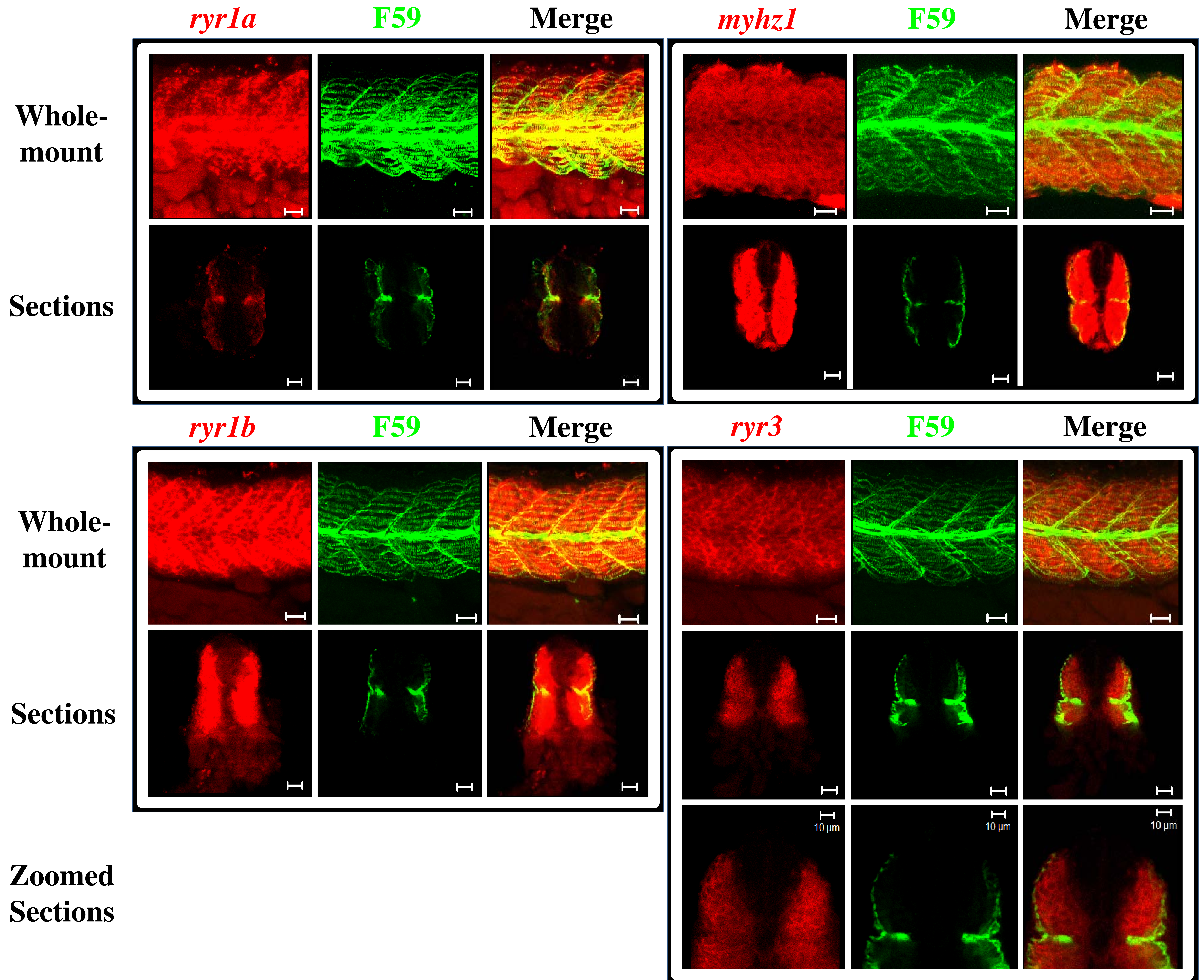

Supplement: Additional file 1 — Figure S1. ryr1a, ryr1b and ryr3 mRNA is differentially expressed in the developing skeletal muscle. Double labelling in wholemount embryos at 24 hpf was performed using probes to myhz1, ryr1a, ryr1b and ryr3 and Fast Red as a substrate (red) followed by immunostaining using the F59 antibody and a fluorescent secondary (green). Images show Z-stacks of whole-mount double-labelled embryos (top row) or sections (bottom row) from dissected embryos. Cross-sectional images revealed that ryr1a co-localised exclusively with slow muscle staining, whereas myhz1, a marker of fast muscle, was not expressed in the slow muscle. Furthermore ryr1b expression could be observed throughout both muscle types whereas ryr3 did not co-localise with the slow muscle staining and appeared to be expressed exclusively in the fast muscle. Scale bars = 20 μm, unless otherwise indicated. [file 1756-0500-4-541-S1.PDF]
