# Supplementary material for: Ryanodine receptors, a family of intracellular calcium ion channels, are expressed throughout early vertebrate development
Source: BMC Res Notes. 2011 Dec 14;4:541. doi: 10.1186/1756-0500-4-541 (PMC3262159; doi:10.1186/1756-0500-4-541)
Supplement: Additional file 2 — Figure S2. ryr3 mRNA expression is confined to the fast muscle fibres throughout the myotome at 24 hpf. Double fluorescent cross-sections (1-3) were prepared by labelling a 24 hpf zebrafish embryo with a fluorescence substrate (i.e. FastRed; red) for ryr3 in situ hybridisation and the F59 antibody (green) for immunostaining. The position of cross-sections 1-3 are illustrated in the 24 hpf ryr3 WISH labelled embryo (top left) which has been stained with BM purple and laterally orientated with anterior to the left. Scale bars = 20 μm, unless otherwise indicated. [file 1756-0500-4-541-S2.PDF]

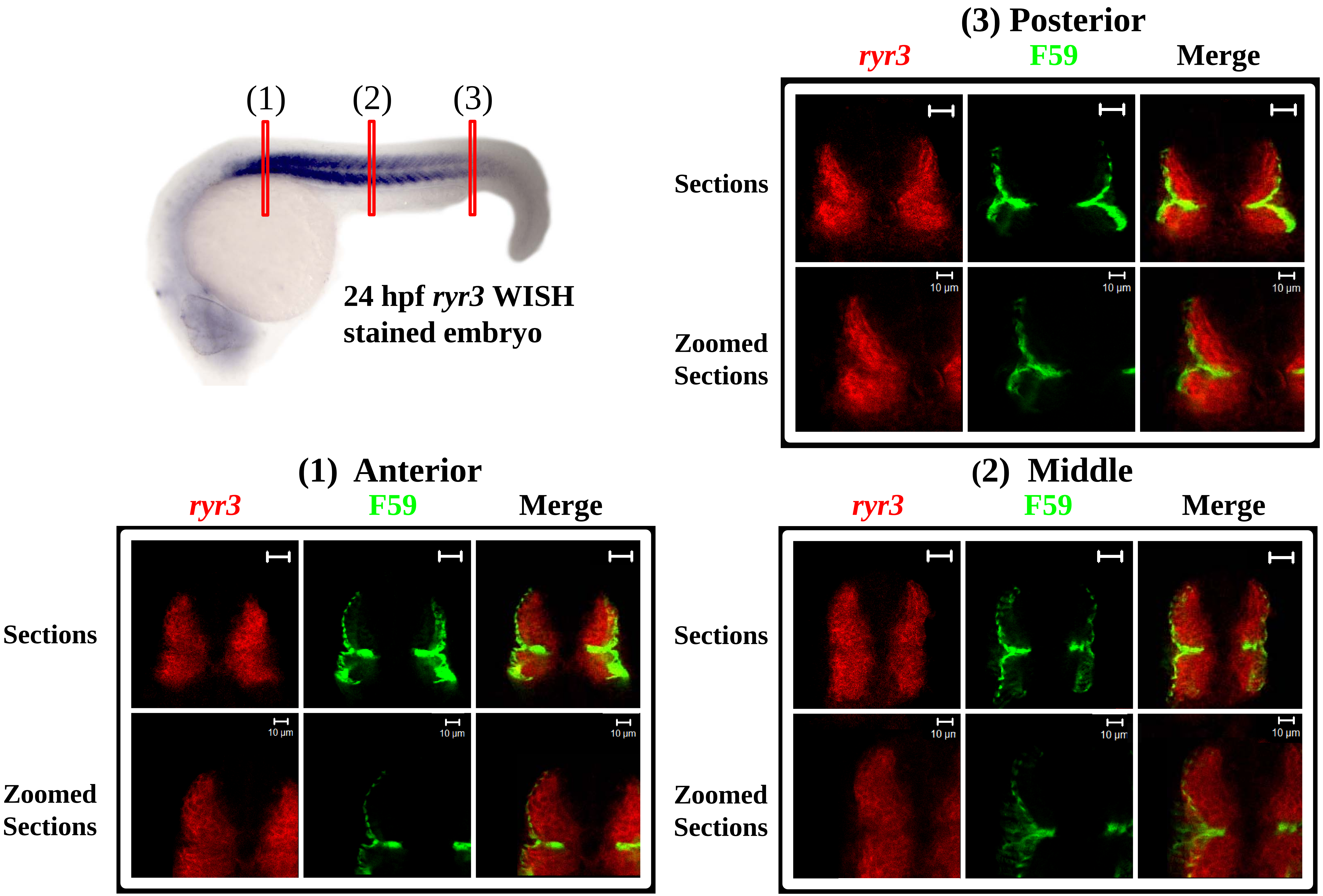

(1)
(2)
(3)
24 hpf ryr3 WISH
stained embryo
(3) Posterior
ryr3
F59
Merge
Sections
Zoomed
Sections
10 µm
(1) Anterior
ryr3
F59
Merge
Sections
Zoomed
Sections
10 µm
(2) Middle
ryr3
F59
Merge
Sections
Zoomed
Sections
10 µm
